# Case report: Whole-exome sequencing for a hereditary elliptocytosis case with an unexpectedly low HbA_1c_

**DOI:** 10.3389/fmed.2023.1301760

**Published:** 2023-12-12

**Authors:** Lu Pang, Ziyi Zeng, Yadi Ding, Haiming Huang, Haixia Li

**Affiliations:** Department of Clinical Laboratory, Peking University First Hospital, Beijing, China

**Keywords:** hereditary elliptocytosis, *SPTB* gene, whole-exome sequencing, glycosylated hemoglobin, case report

## Abstract

**Objectives:**

Hereditary elliptocytosis is a group of erythroid hereditary diseases characterized by elliptically shaped erythrocytes in peripheral blood. It is mainly inherited through autosomal dominant inheritance. This study aimed to conduct a genetic etiology analysis in a case with a clinical diagnosis of hereditary elliptocytosis and an unexpectedly low HbA_1c_.

**Methods:**

Whole-exome sequencing was performed to find the possible pathogenic mutations. At the same time, bioinformatics software was used to predict the mutation function. Sanger sequencing was performed to verify the suspected pathogenic mutations.

**Results:**

Whole-exome sequencing results showed that the proband with mild anemia had a heterozygous c.2303G>A (p.G768D) missense mutation in the 13th exon of the *SPTB* gene. The Sanger sequencing confirmed this heterozygous mutation. This mutation was extremely rare in the population, and multiple software’s predictions were harmful. Conservative analysis revealed that this site was highly conserved in various species.

**Conclusion:**

The c.2303G>A mutation of the *SPTB* gene is the suspected cause of hereditary elliptocytosis in the patient. Our data show that microscopic examination of red blood cells on blood smears is an important means of diagnosing hereditary elliptocytosis. Whole-exome sequencing is an effective tool to determine the genetic etiology of erythrocyte membrane diseases, which can promote accurate diagnosis and genetic counseling.

## 1 Introduction

Hereditary elliptocytosis (HE) is a disease characterized by an increase in elliptical red blood cells caused by abnormalities in the cytoskeletal proteins of the red blood cell membrane (1). On the blood smear, abnormal red blood cells such as oval-shaped red blood cells, rod-shaped red blood cells, and red blood cell fragments are present. The prevalence of HE in the world is approximately 1:2,000–4,000, but the prevalence is higher in some African regions (1,100) (2). This disease is mainly inherited in an autosomal dominant manner (2, 3). HE is mainly caused by mutations in genes encoding red blood cell membranes or skeleton proteins, which disrupt the horizontal interaction between membrane skeleton proteins and cell membranes in the polymer, leading to a decrease in red blood cell deformability and changes in cell membrane function. The main pathogenic genes include *SPTA1* (65%), *SPTB* (30%), and *EPB41* (5%) (4–6). The clinical phenotype of HE patients is highly heterogeneous. They can be divided into asymptomatic type, hemolytic compensatory type, and hemolytic anemia type (7).

Glycated hemoglobin A_1c_ (HbA_1c_), which can reflect the average blood glucose level in the past 8–12 weeks, is recommended both for glycemic control and the diagnosis of diabetes mellitus. Therefore, the interpretation of HbA_1c_ results is closely related to clinical diagnostic strategies (8). The diagnosis of HE mainly depends on the medical history, peripheral blood smear, sodium dodecyl sulfate polyacrylamide gel electrophoresis, etc. Due to the significant heterogeneity of clinical manifestations, patients are prone to missed diagnoses based solely on the above tests. With the advent of high-throughput sequencing, it can improve the diagnostic rate and study pathogenesis at the molecular level. At present, the study of HE has rarely been reported in China. The pathogenesis and mutations of HE vary among different regions and races. Moreover, no heat mutation sites have been found yet. This study conducted molecular diagnostic research on a HE patient with a low value of HbA_1c_ and discovered a rare pathogenic mutation of the *SPTB* gene in China, providing a basis for clinical diagnosis and genetic counseling for HE.

### 1.1 Methods

This study has been approved by the Ethics Committee of Peking University First Hospital. Written informed consent to participate in this study was provided. Genomic DNA was extracted using the QIAamp DNA extraction kit. The extracted DNA was fragmented by DNase and purified using the magnetic bead method. Subsequently, PCR amplification and adaptor sequence connections were performed. The exon region was captured using a commercial Agilent SureSelect Human All Exon V6 reagent kit capture probe, and the final library was sequenced on the Illumina PE150 platform. The average sequencing depth of the target area was 139.00 X. The average Q30 was 93.77%. The effective sequencing data were mapped to the reference genome (GRCh37/hg19) using BWA. Using the literature reporting method, we annotated the data (9). Based on the candidate mutations obtained from the above analysis process, primers were designed for the nearby sequences of the *SPTB* (NM_001024858) mutation. The primers were as follows: upstream, 5′-GCATAAAGGAGGTGTGTGCGGCA-3′; downstream, 5′-CCTGTGGTAGAGCCCCG-3′, with an amplification product length of 348 bp. The PCR amplification product was subjected to sequencing using the ABI 3500.

### 1.2 Case presentation

**Figure 1:**
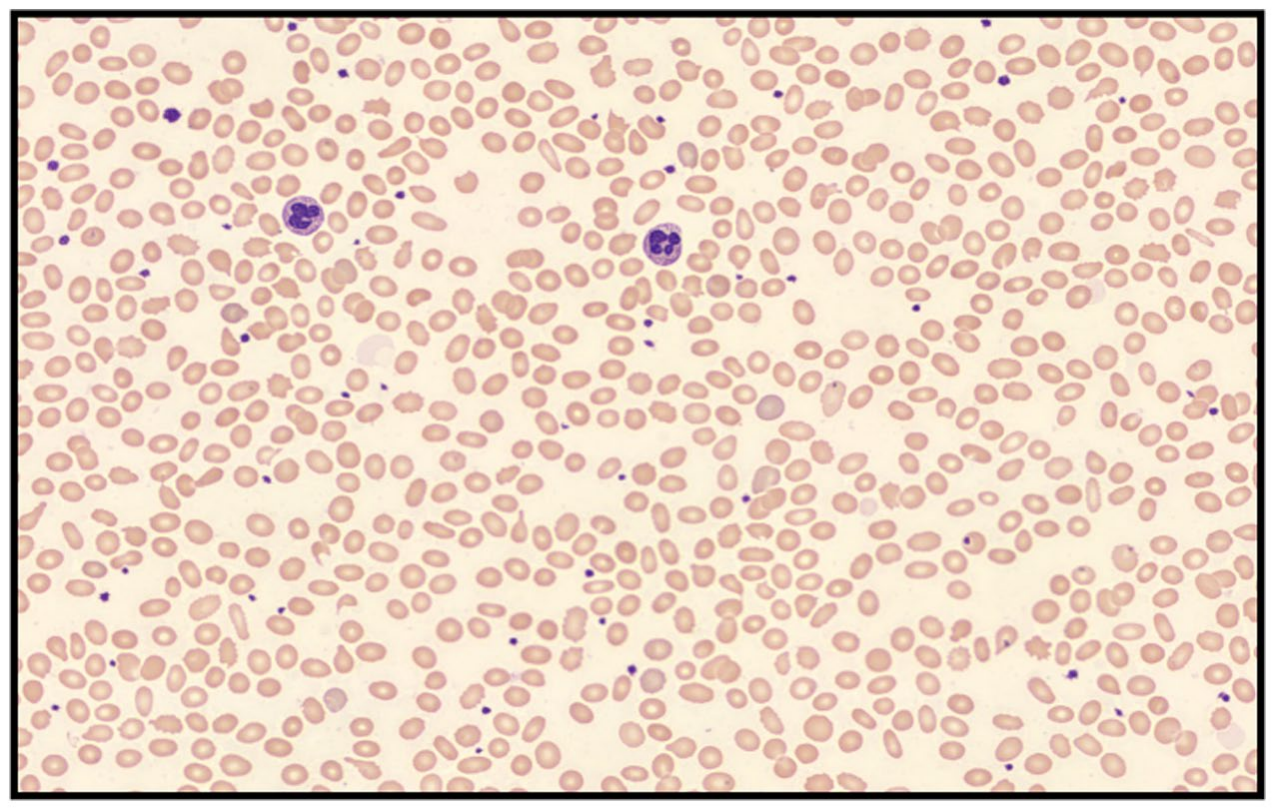
Peripheral blood smear revealed elliptocytes with varying cell sizes that appeared as teardrops, fragments, and rod-shaped forms (Wright-Giemsa stained; 400 × magnification).

A 45-year-old man came to our hospital for a routine health examination and had an unexpectedly low HbA_1c_ concentration of 3.9%. The results of fasting blood glucose and glycated albumin were normal. The chromatogram showed no abnormalities, thus excluding interference from hemoglobin variants. The blood routine results indicated a decrease in the number of red blood cells and hemoglobin concentration and an increase in reticulocyte count. Biochemical examination indicated a slight increase in direct bilirubin (Table 1). Mild hemolysis was present in the serum. These results supported the presence of hemolytic anemia in patients. The blood smear result indicated that the patient had an increase in oval-shaped red blood cells with varying cell sizes. Teardrops, fragments, and rod-shaped red blood cells were visible (Figure 1). In addition, the patient’s Coombs test was negative.

**Table 1:** Laboratory findings and clinical traits of the patient.

Characteristics	Results	Reference range
WBC, *10^9^/L	5.4	3.5–9.5
RBC, *10^12^/L	3.4	4.3–5.8
Hemoglobin, g/L	109.0	130.0–175.0
RET, %	2.83	1.0–2.5
RET, *10^9^/L	97.1	24.0–84.0
RDW, %	18.5	<14.9
DBIL, μmol/L	7.0	0–6
TBIL, μmol/L	30.9	1.7–20

WBC, white blood cells; RBC, red blood cells; RET, reticulocyte; RDW, red cell distribution width; DBIL, direct bilirubin; TBIL, total bilirubin.

**Figure 2:**
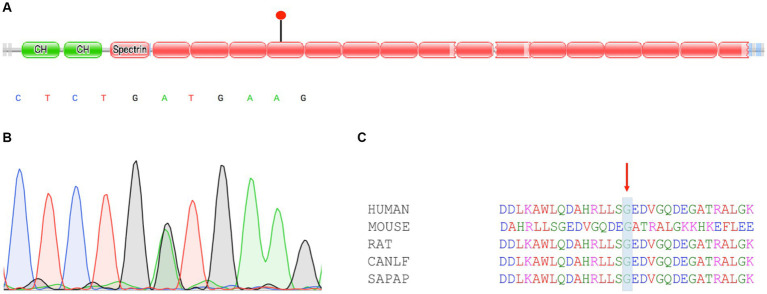
Characteristics and validation of *SPTB* mutation. **(A)** Diagrammatic representation of SPTB with known protein domains. The mutation c.2303G>A (p.G768D) found in this study had been indicated. **(B)** Sanger sequencing confirmed *SPTB* mutation. **(C)** Multiple sequence alignment of homologs from different species. Conservative regions were marked.

We learned that both the patient’s father and uncle had a family history of anemia; thus, whole-exome sequencing (WES) was used to detect gene mutations in this patient. A heterozygous missense mutation c.G2303A (p.G768D) was detected in the *SPTB* gene (Figure 2A). We validated the suspicious mutation of the *SPTB* gene using Sanger sequencing, and the results were consistent with high-throughput sequencing (Figure 2B). This mutation is extremely rare in the population, with a minor allelic frequency (MAF) of <1% in gnomAD. Multiple software programs predict this mutation harmfully. This missense mutation affects the self-association of spectrin proteins. This mutation site is highly conserved in function and evolution among different species (Figure 2C). Unfortunately, the patient’s father has passed away, and his uncle’s sample was not obtained.

## 2 Discussion

HE is a group of hereditary hemolytic diseases characterized by an increase in elliptical red blood cells in the peripheral blood. The typical clinical manifestation of HE includes varying degrees of anemia, intermittent jaundice, and splenomegaly. Due to the high clinical heterogeneity of HE, it is easy to be missed or misdiagnosed. Understanding HE from clinical characteristics and etiology is of great significance for clinical treatment management. This study revealed a rare case of HE caused by an *SPTB* mutation in a health examination with a low value of HbA_1c_ in China, providing new data for the genetic etiology of HE and promoting research on the mechanism of HE.

Hemolytic anemia is a group of anemia that occurs when the destruction and growth rate of red blood cells exceed the compensatory capacity of hematopoiesis. It is the most common disease that shortens the lifespan of red blood cells, leading to a decrease in HbA_1c_ levels. When a patient exhibits hemolytic anemia, such as a decrease in red blood cell count and hemoglobin and an increase in direct bilirubin, HbA_1c_ levels will significantly decrease. The red blood cell membrane is composed of a lipid bilayer and proteins distributed within it. The complete membrane protein interaction, responsible for the strength and deformability of red blood cells, can maintain the double concave disk structure of red blood cells (1). The normal membrane skeleton protein is mainly composed of spectrin protein, 4.1 protein, and actin. Many hereditary hemolytic anemias are caused by genetic mutations that cause abnormalities in red blood cell membrane proteins or quantities. According to literature reports, the pathogenesis of HE at the molecular level is mainly due to mutations in the *SPTA1*, *SPTB*, and *EPB41* genes, which encode α spectrin protein, β spectrin protein, and 4.1 protein, respectively (10). These mutations cause the instability of the surface skeleton of the elliptical red blood cell membrane, which is then blocked in the spleen system, resulting in abnormal manifestations such as hemolysis and increased bilirubin.

Family studies have shown that genetic factors play an important role in the pathogenesis of HE (1). The *SPTB* gene is located at 14q23.3 and contains 38 exons encoding 2,137 amino acids. The *SPTB* gene is composed of three structural domains: Domain I is a 272 amino acid region at the N-terminus; Domain II consists of 17 spectral repeat sequences; Domain III, with 52 amino acid residues at the C-terminus, does not adhere to the spectral repeat motif (11). A total of 30% of HE patients are caused by β-spectrin protein mutations. Homozygous mutations can cause severe hemolysis or even death in patients, while the clinical manifestations of heterozygous mutations are diverse, and fetal edema can also occur in severe cases (12). *In vitro* studies have found that missense mutations and fragment deletions at the carboxyl end of β spectrin protein can directly affect the self-association of spectrin protein, and mutations in this region are related to the heterogeneity of clinical manifestations in HE (13). Christensen et al. reported a case of persistent jaundice in a newborn with the *SPTB* gene combined with the *SPTA1* gene mutation. The child had a clear family history of HE, but no significant hemolysis or jaundice was observed in family members. This type of newborn should be carefully examined for the morphology of red blood cells for a clear diagnosis (14). Currently, genetic research in HE cases has not attracted sufficient attention in China. Wang et al. reported a HE case with variable expression and incomplete penetrance in a Chinese family. The heterogenous *SPTA1* IVS33-1G>A mutation was responsible for HE (15). Cao et al. identified a heterozygous mutation 1294delA in exon 15 of the *EPB41* gene using high-throughput sequencing and Sanger sequencing in members of a Chinese family (16).

In addition, HE clinically can be classified into asymptomatic type, hemolytic compensatory type, and hemolytic anemia type based on the patient’s hemoglobin, reticulocyte count, jaundice, and degree of splenomegaly (7). Asymptomatic and hemolytic compensatory types generally have no clinical signs such as anemia and jaundice, and there may even be no abnormal red blood cells in peripheral blood smears. Patients with hemolytic anemia often experience a decrease in hemoglobin, an increase in reticulocytes, and an increase in abnormal red blood cells in the peripheral blood. In this study, the proband presented with mild anemia, with an increased proportion of reticulocytes, elevated levels of bilirubin in the blood, and elliptical red blood cells easily visible in peripheral blood smears. He was classified as having a hemolytic anemia type. The lifespan of HbA_1c_ is consistent with that of red blood cells. Any factor that can shorten the lifespan of red blood cells, reduce the exposure time of red blood cells in high glucose environments, or increase red blood cell turnover can cause a decrease in HbA_1c_ levels. Patients with hematological diseases, especially those with hemolytic anemia, have a shorter lifespan of red blood cells, resulting in a relatively shorter time for the formation of glycated hemoglobin. Therefore, the HbA_1c_ result will be lower (17, 18).

Molecular genetics technology is a progressive technology to determine gene mutations. Combined with multiple laboratory tests, it is conducive to improving the efficiency of the HE diagnostic level. Molecular diagnosis can identify genotype–phenotype correlations of these heterogeneous diseases and may aid in disease prognosis (19). The Sanger sequencing method can directly obtain information about mutation sites, which has advantages in detecting unknown mutations. However, in severe transfusion-dependent cases, multiple sources of red blood cell populations in the body can disrupt their detection results. The high-throughput sequencing technology can provide efficient and rapid diagnosis for hereditary hemolytic diseases, and even in patients with multiple blood transfusions. Lacy et al. reported a case with severe hemolysis and multiple blood transfusions in HE patients. Finally, a high-throughput sequencing test revealed a mutation in the 4.1 protein, confirming the diagnosis of HE (20).

Our research expands our insights into the molecular epidemiology of red blood cell membrane diseases in East Asia. Our research also has some shortcomings; further functional studies, such as cell experiments or animal studies, may be needed to validate the pathogenicity of this mutation. The next research is to observe whether the mutation has an impact on red blood cell deformability and the number of β-spectrin-targeted erythrocyte membranes. In addition, additional research in a larger cohort should be conducted to construct a genetic map of HE in patients with red blood cell membrane disease in China. The results of this study and other studies indicate that high-throughput sequencing is a powerful tool for identifying single nucleotide variations in HE, but the large number of mutations identified in sequencing is also a huge challenge. Therefore, there is a need for larger population validation or validation in animal models, or it is possible to conduct such studies on a large number of trios. As more and more HE patients undergo comprehensive sequencing assessments, it is more likely to identify the causes of most HE and translate these findings into precision medicine.

## 3 Conclusion

In summary, this study identified a rare pathogenic mutation in the *SPTB* gene in China through high-throughput sequencing, which has not been reported in previous studies. The results of this study extend the mutation spectrum of the *SPTB* gene in HE patients and provide a reference for further exploration of the genetic causes of HE in Chinese. Our data also show that microscopic examination of red blood cells on blood smears is an important means of diagnosing hereditary elliptocytosis. Whole-exome sequencing is an effective tool to determine the genetic etiology of erythrocyte membrane diseases, which can promote accurate diagnosis and genetic counseling.

On the one hand, samples with abnormal glycosylated hemoglobin can be collected in future and their etiology may be diagnosed through high-throughput sequencing. On the other hand, functional validation is required for the discovered HE mutations.

## Data availability statement

The datasets presented in this article are not readily available because of ethical/privacy restrictions. Requests to access the datasets should be directed to the corresponding author.

## Ethics statement

The studies involving humans were approved by the Ethics Committee of Peking University First Hospital. The studies were conducted in accordance with the local legislation and institutional requirements. The participants provided their written informed consent to participate in this study. Written informed consent was obtained from the individual(s) for the publication of any potentially identifiable images or data included in this article.

## Author contributions

LP: Conceptualization, Data curation, Supervision, Writing – review & editing. ZZ: Data curation, Formal analysis, Investigation, Writing – review & editing. YD: Investigation, Writing – original draft. HH: Investigation, Writing – original draft, Conceptualization. HL: Supervision, Writing – review & editing.
